# Novel *Methanobacterium* Strain Induces Severe Corrosion by Retrieving Electrons from Fe^0^ under a Freshwater Environment

**DOI:** 10.3390/microorganisms10020270

**Published:** 2022-01-25

**Authors:** Shin-ichi Hirano, Sota Ihara, Satoshi Wakai, Yuma Dotsuta, Kyohei Otani, Toru Kitagaki, Fumiyoshi Ueno, Akihiro Okamoto

**Affiliations:** 1 Central Research Institute of Electric Power Industry (CRIEPI), Chiba 270-1166, Japan; 2 Graduate School of Science and Technology, University of Tsukuba, Ibaraki 305-8573, Japan; souta990209@outlook.jp; 3 Institute for Extra-Cutting-Edge Science and Technology Avant-Garde Research (X-Star), Japan Agency for Marine-Earth Science and Technology (JAMSTEC), Kanagawa 237-0061, Japan; wakais@jamstec.go.jp; 4 Collaborative Laboratories for Advanced Decommissioning Science, Japan Atomic Energy Agency (JAEA), Ibaraki 319-1106, Japan; dotsuta.yuma@jaea.go.jp (Y.D.); otani.kyohei@jaea.go.jp (K.O.); kitagaki.toru@jaea.go.jp (T.K.); ueno.fumiyoshi@jaea.go.jp (F.U.); 5 International Center for Materials Nanoarchitectonics, National Institute for Materials Science (NIMS), Ibaraki 305-0044, Japan; OKAMOTO.Akihiro@nims.go.jp; 6 Graduate School of Chemical Sciences and Engineering, Hokkaido University, Sapporo 060-8628, Japan

**Keywords:** methanogen, iron corrosion, extracellular electron uptake

## Abstract

Methanogens capable of accepting electrons from Fe^0^ cause severe corrosion in anoxic conditions. In previous studies, all iron-corrosive methanogenic isolates were obtained from marine environments. However, the presence of methanogens with corrosion ability using Fe^0^ as an electron donor and their contribution to corrosion in freshwater systems is unknown. Therefore, to understand the role of methanogens in corrosion under anoxic conditions in a freshwater environment, we investigated the corrosion activities of methanogens in samples collected from groundwater and rivers. We enriched microorganisms that can grow with CO_2_/NaHCO_3_ and Fe^0^ as the sole carbon source and electron donor, respectively, in ground freshwater. *Methanobacterium* sp. TO1, which induces iron corrosion, was isolated from freshwater. Electrochemical analysis revealed that strain TO1 can uptake electrons from the cathode at lower than −0.61 V vs SHE and has a redox-active component with electrochemical potential different from those of other previously reported methanogens with extracellular electron transfer ability. This study indicated the corrosion risk by methanogens capable of taking up electrons from Fe^0^ in anoxic freshwater environments and the necessity of understanding the corrosion mechanism to contribute to risk diagnosis.

## 1. Introduction

Iron (Fe^0^) is used in steel structures in many industrial processes, such as oil, chemical, and gas production, and the electric power industry (e.g., transport pipelines, cooling circuits, storage, water tanks, underground storage, reactors, and parts of plant facilities). Corrosion can cause severe economic damage to steel infrastructure. The estimated global annual cost of corrosion in 2013 was USD 2.5 trillion, which is 3.4% of the gross domestic product of industrialized countries [1]. Iron corrosion is induced by an electrochemical reaction of iron oxidation and dissolution (anodic reaction) and electron uptake by external electron acceptors (cathodic reaction); see Equation (1). Under aerobic conditions, oxygen is the main acceptor in cathodic reactions. In the absence of oxygen, reducing protons (H_2_ evolution) can work for a cathodic reaction; see Equation (2). Therefore, protons can oxidize Fe^0^, and molecular hydrogen is generated by an exergonic reaction. Since proton reduction on iron surfaces is significantly slow under neutral pH due to the low availability of protons, metallic iron is usually stable under anoxic conditions. However, microorganisms can increase corrosion and have severe effects under anoxic conditions [2,3]. Microbially influenced corrosion (MIC) accounts for 10% of all corrosion damage to steel structures in contact with salty water or freshwater containing various microorganisms [4,5]. Therefore, it is important to develop effective monitoring and preventative methods to minimize the economic losses and environmental pollution caused by MIC damage. However, MIC-related processes and mechanisms are not sufficiently understood.

To date, two MIC mechanisms have been proposed. First, metabolites of microorganisms indirectly induce corrosion, designated as chemical MIC (CMIC) (e.g., H_2_S produced by sulfate-reducing bacteria (SRB)) [6,7]. Second, microorganisms directly induce corrosion by taking up cathodic electrons from the iron surface using specific enzymes, designated as electrical MIC (EMIC) [7]. EMIC causes progressive corrosion of metallic iron without a significant slowdown due to the crust formation of corrosion products, in contrast to the corrosion caused by CMIC. A limited number of EMIC microbes have ben currently identified. Dinh et al. isolated a novel SRB (*Desulfopila corrodens* strain IS4, *Desulfovibrio ferrophilus* IS5) and a methanogenic archaea (*Methanobacterium* sp. strain IM1) from anoxic marine sediment that used Fe^0^ as the sole electron donor [4]. EMIC isolates take up electrons in conjunction with sulfate reduction and methanogenesis; see Equations (3) and (4) [4,8]. Since these conjunctions are thermodynamically probable, it has been proposed that SRB and methanogen can grow using Fe^0^ as an electron donor by exergonic reaction. SRB with EMIC activity were investigated in previous studies, and multi-heme cytochromes were identified as enzymes responsible for electron uptake from iron [9,10,11]. The EMIC methanogens, *Methanococcus maripaludis* and *Methanosarcina* strains, that acquire electrons directly from iron were isolated from a high-salinity environment [12,13]. *M. maripaludis* possesses [NiFe] hydrogenase for direct electron uptake from the iron surface, encoded by an unstable genomic region with an enzyme secretion system [14]. Lahme et al. performed successful PCR amplification of genes similar to those encoding [NiFe] hydrogenase in several oil-field samples where corrosion activity was observed, thus, indicating that the mechanism is widespread in marine microbes [15]. EMIC-inducing methanogens have been isolated from the marine environment. However, there are no reports of methanogenic isolates with EMIC activity in freshwater environments. A freshwater environment is suitable for the growth of methanogens without sulfate or electron acceptor, except for CO_2_. Therefore, to understand the role of methanogens in corrosion under anoxic conditions in freshwater, we investigated the corrosion activities of methanogens in samples collected from groundwater and rivers. We enriched and isolated a novel methanogen capable of utilizing Fe^0^ as the sole electron donor in a freshwater environment. The isolate was then electrochemically analyzed to evaluate its EMIC activity.

Fe^0^ → Fe^2+^ + 2e^−^, *E_0′_*= −0.47 V (vs. SHE) (1)

2H^+^ + 2e^−^→ H_2_; *E_0′_*= −0.41 V (vs. SHE) (2)

SO_4_^2−^ + 8e^−^ + 9H^+^ → HS^−^ + 4H_2_O; *E_0′_*= −0.22 V (vs. SHE) (3)

HCO_3_^−^ + 9H^+^ +8e^−^ → CH_4_ + 3H_2_O; *E_0′_*= −0.24 V (vs. SHE) (4)

## 2. Materials and Methods

### 2.1. Site and Sample Description

**Figure 1:**
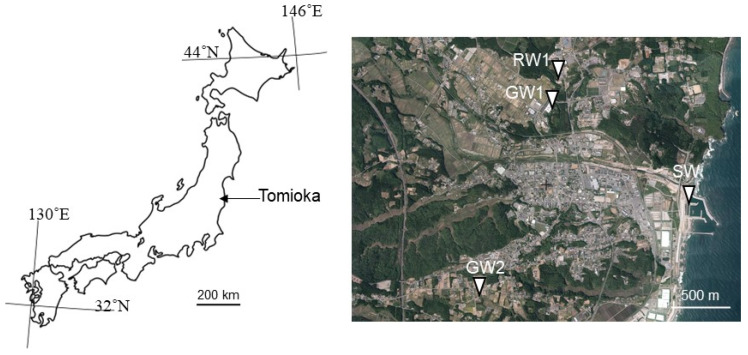
Sampling points in this study. This map is based on the digital map published by the Geospatial Information Authority of Japan.

Sampling was carried out from sites around Tomioka, Fukushima, Japan (Figure 1). Two samples of groundwater (GW1 and GW2) were collected from wells and spring ponds. River water (RW) was collected from a tributary of the Tomioka river. Water samples were collected using sterilized polypropylene bottles (1 L volume) on 17 November 2020 and stored at 4 °C until further analysis. Water pH and oxidation-reduction potential (ORP) were measured in the field using portable meters (WQ-320J, HORIBA, Kyoto, Japan) at four points, as shown in Figure 1.

### 2.2. Analytical Methods

Chemical analysis of the ground, river, and seawater samples filtered through a 0.45 µm filter membrane (ToyoRoshi Kaisha, Tokyo, Japan) was performed by ion chromatography ICS-1500 equipped with AS11-HC and an ICS-2000 equipped with CS16A (Dionex, Sunnyvale, CA, USA) to determine the presence of Cl^–^, NO_2_^–^, NO_3_^–^, Br^–^, SO_4_^2–^, Li^+^, Na^+^, NH_4_^+^, K^+^, Mg^2+^, and Ca^2+^. In addition, the ferrous iron levels in the water samples were measured as previously described [16].

### 2.3. Cultivation Methods for Enrichment and Isolation of Microorganisms with EMIC Activity

#### 2.3.1. Enrichment Method of Iron-Corroding Microorganisms from Collected Samples

Collected water samples were cultivated using serum bottles (50 mL) filled with 30 mL of a freshwater basal (FB) medium containing the following ingredients (per liter): 23.8 g of 4-(2-hydroxyethyl)-1-piperazineethanesulfonic acid; 2.6 g of MgCl_2_·6H_2_O; 0.15 g of CaCl_2_·2H_2_O; 4.0 g of Na_2_SO_4_; 0.25 g of NH_4_Cl; 0.2 g of KH_2_PO_4_; 0.5 g of KCl; 2.52 g NaHCO_3_; 1 g of cysteine-HCl; and 1 mL each of trace metal solution and vitamin solution. The pH of the medium was adjusted to 7.0 [12]. In addition, 2 g of iron granules (1–2 mm in diameter, 99.98% purity, Tewksbury, MA, Alfa Aesar), autoclaved under N_2_ conditions, was supplemented to FB medium as the sole electron donor. After sealing the serum bottles, the headspace was repeatedly replaced by vacuuming out and charging an N_2_/CO_2_ gas mixture at a 4:1 (*v*/*v*) ratio. Then, collected water samples (3 mL) were inoculated as a source of microorganisms, and the inoculated bottles were incubated at 30 °C without shaking. All chemicals were purchased from Fujifilm Wako Pure Chemical Co. (Osaka, Japan).

To evaluate the metabolic activity after 1 month or 3 months of cultivation, the H_2_ and CH_4_ contents in the headspace were measured using a gas chromatograph equipped with a thermal conductivity detector (CP400; Varian Medical Systems, Palo Alto, CA, USA) and a Molsieve 5A column (GL Sciences Inc., Tokyo, Japan) at 100 °C using Ar as a carrier gas [17]. The Fe^2+^ concentration dissolved in the culture due to corrosion was measured by colorimetric method as previously described [16]. Cultures showing corrosion activity were transferred to freshly prepared FB medium containing iron granules after brief homogenization.

#### 2.3.2. Isolation of Methanogens with EMIC Activity

Enriched culture with corrosion activity after multiple transfers in the presence of iron granules was used to isolate microorganisms responsible for EMIC. Enriched culture after brief homogenization to detach cells from iron granules was spread on agar plates of FB medium solidified with 1.5% (wt/vol) agarose XP (Fujifilm Wako Pure Chemical Corp., Osaka, Japan) in an anaerobic chamber (Coy Laboratory Products, Inc., Grass Lake, MI, USA) filled with N_2_ gas. The inoculated agar plates were incubated in an AnaeroPouch (Mitsubishi Gas Chemical Corp. Inc., Tokyo, Japan) filled with H_2_/CO_2_ (4:1 *v*/*v*) at 30 °C. Colonies grown on agar plate were transferred to newly prepared FB liquid medium with iron granules and corrosion activity was examined. Furthermore, culture with corrosion activity was conducted by repetitive colony isolation. The purity of the isolated colony was confirmed by molecular and microscopic analyses. 

To measure corrosion activity, the isolated methanogen was cultivated in a FB liquid medium with a carbon steel coupon as the sole electron donor, instead of iron granules. The surface of a carbon steel SS400 sheet (0.5 mm-thick) was polished using no. 120 and no. 400 paper (MonotaRO Co., Ltd., Hyogo, Japan). A polished carbon steel sheet was cut into 8 × 8 mm coupons, and these coupons were cleaned in acetone. After drying, initial weights of carbon steel coupons were measured. Each coupon was separately autoclaved in a closed serum bottle filled with N_2_ condition at 121 °C for 15 min and stored before use. A sterilized coupon was placed on the bottom of a 50 mL serum bottle and filled with 30 mL of FB liquid medium. After sealing the bottles and repeated gas replacement in the headspace with N_2_/CO_2_ gas (4:1, *v*/*v*), the bottles were incubated at 30 °C without shaking for 2 months. After 2 months, the headspace of the serum bottle was analyzed by gas chromatography and the bottle was opened to pick up the carbon steel coupons. The carbon steel coupons were weighed after removing corrosion products formed on the coupon surface with Clark’s solution (1000 mL hydrochloric acid, 20 g Sb_2_O_3,_ and 50 g SnCl_2_), according to ASTM G1 (ASTM, 2011). The weight loss of the carbon steel coupon due to corrosion was calculated by comparing the initial weight with the weight after incubation and removing corrosion products. Abiotic controls were also prepared and incubated using the same method but without inoculation. After incubation, the weight loss of abiotic control was calculated using the same method as inoculated samples. The data are presented as the mean and standard deviation of three independent replicates. The average corrosion rates (mm/yr), in culture of isolated methanogen or abiotic control, were calculated as follows, in Equation (5):

Corrosion rate = (K × W)/(A × T × D)(5)

where K is a constant (365 × 10), W is the weight loss of coupon (mg), T is the incubation time (days), A is the surface area of coupon (cm^2^), and D is the density of iron (g/cm^3^).

Genomic DNA was extracted from the culture of isolated methanogen by using DNeasy PowerSoil Pro Kit (QIAGEN, Venlo, Netherland). The 16S rRNA gene of isolated methanogen was amplified using extracted genomic DNA with primer sets, Arch f2/Arc r1386 for, as previously described [18]. The amplified 16S rRNA gene was sequenced using the Sanger sequencing method (performed at FASMAC Co., Ltd., Kanagawa, Japan). The identified sequence of the isolate was deposited in DNA Data Bank of Japan under accession number LC664176. The related methanogens to the obtained sequence of the isolate were estimated by using the BLASTn algorithm against the sequences in the GenBank database available from the National Center for Biotechnology Information. Alignment and phylogenetic reconstructions of strain TO1 among other species of *Methanobacterium* and *Methanothermobacter thermoautotrophicus* strain delta H (NR_042782) as an out-group were performed using the function “build” of ETE3 v3.1.1 [19] as implemented on the GenomeNet (https://www.genome.jp/tools/ete/ (accessed on 28 December 2021)). A phylogenetic tree was constructed using FastTree [20].

### 2.4. Electrical Incubation and Cyclic Voltammetry Measurement of Cells

The electrical incubation was conducted in a three-electrode reactor in an anaerobic chamber filled with 95% N_2_ and 5% H_2_. NBRC 927 medium without NaCl, Na_2_SO_4_, iron granule (1–2 mm diameter), or cysteine-HCl was used as the electrolyte. The same solution without NaHCO_3_ was used as the negative control. NaHCO_3_ is ionized into Na^+^ and HCO_3_^–^ in neutral water and HCO_3_^−^ act as a sole electron acceptor for methanogen; see Equation (4) [8]. In addition, we used a screen-printed electrode system with carbon electrodes as the working electrode (surface area, 7.07 mm^2^) and the counter electrode. Ag/AgCl was used as the reference electrode. All electrodes were placed at the bottom of the reactor. In the presence of *Methanobacterium* cells at 600 nm of 0.5, electrical incubation was initiated with electrode potentials poised at −0.23, −0.42, −0.61, and −0.83 V vs. a standard hydrogen electrode (SHE). Cyclic voltammetry (CV) measurements were conducted at a scan rate of 1 mV/s from −1.03 to 0.173 vs. SHE. Conversion to SHE was performed to compare the potential difference between the Ag/AgCl printed electrode and Ag/AgCl electrode in KCl saturated solution using a digital multimeter immediately after performing each electric incubation and CV. In all the measurements, the potential shift was less than 2.3 mV.

## 3. Results

### 3.1. Water Chemistry

Groundwater (GW1 and GW2), river water (RW), and seawater (SW) were collected from Tomioka, Fukushima, Japan. The bulk water chemistry data are shown in Table 1. The freshwater samples had low SO_4_ concentrations of approximately 10 ppm. The two groundwater samples (GW1 and GW2) showed chemical profiles with small differences. GW1, RW, and SW samples had a higher pH (around pH 8.0), compared to the GW2 sample (pH 7.0). The GW1 sample had a slightly higher concentration of the main components, such as Cl^−^, Na^+^, Ca^2+^, K^+^, and Mg^2+^, than the GW2 sample. The GW1 sample was presumed to be affected by water that was sustained underground for a more extended period than GW2 sample. 

### 3.2. Enrichment and Isolation of Microorganisms with EMIC Activity

#### 3.2.1. Enrichment of Iron-Corroding Microbial Communities

To enrich microorganisms that induce iron corrosion, the collected water samples were inoculated in freshwater basal salt medium containing NaHCO_3_/CO_2_ as the sole carbon source and supplemented with iron granules as the sole electron donor under N_2_/CO_2_ gas. After 1 month of cultivation, black precipitates were formed on iron granules in the enrichment culture with GW2 as an inoculation source (designated as e-GW2). At the same time, there were no changes in the abiotic control (without inoculation) and other samples (e-GW1, e-RW, and e-SW). Two of five replicates of the e-GW2 sample showed a decrease in sulfate ion (2600 ppm to 2223 ppm) concentration and an increase in Fe^2+^ concentration (3.7 mM to 4.0 and 4.6 mM) by corrosion in the medium. H_2_ and CH_4_ were not observed in the headspace of the e-GW2 sample. It was estimated that the cultures included SRB that can grow autotrophically using iron granules as the electron donor. In contrast, after continuous cultivation for 3 months, a grayish corrosion product was found on the iron granules in one of the five cultures of the e-GW1 sample. In this culture, CH_4_ generation and high Fe^2+^ concentration in the medium (9.8 mM) were detected, suggesting that corrosive methanogens grew in the e-GW1 sample. After 3 months, e-RW and e-SW samples showed no corrosion activity. Therefore, active e-GW1 and e-GW2 cultures were transferred to freshly prepared freshwater basal medium and enriched for corrosive microorganisms with the ability to use iron granules as the sole electron donor.

**Figure 2:**
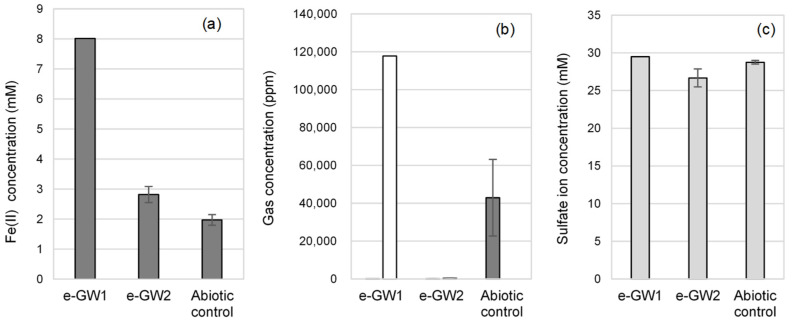
Corrosion activity in enriched cultures after 2 months of cultivation. (**a**) Fe(II) concentration after iron dissolution by corrosion, (**b**) gas contents in the headspace (gray bar (CH_4_), white bar (H_2_)), and (**c**) sulfate ion concentration in culture medium.

**Figure 3:**
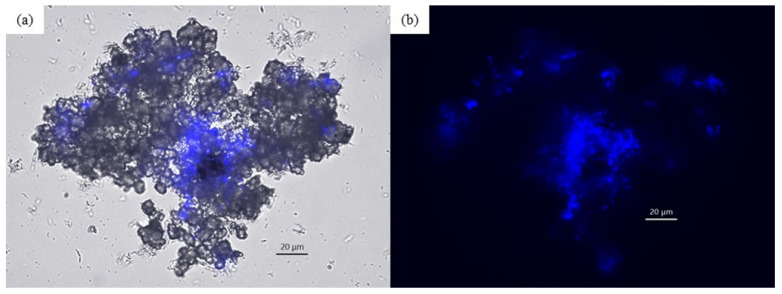
Microscopic observation of enriched culture (e-GW1) overlayed with auto-fluorescence of methanogens (**a**) and auto-fluorescence of methanogens (**b**).

After multiple subcultures, the corroding activities of the e-GW-1 and e-GW2 cultures were measured and compared with that of the abiotic control after 1 month of cultivation (Figure 2). The Fe^2+^ concentration was four times higher in the e-GW1 culture and 1.5-fold higher in the e-GW2 culture than that in the abiotic control. The analysis of gas-phase components showed H_2_ generation in the abiotic control due to abiotic iron corrosion (42,945 ppm), but H_2_ could not be detected in e-GW1 and e-GW2 cultures. Methane generation was observed only in the e-GW1 culture (117,753 ppm). Fluorescence microscopic observation revealed that rod-shaped cells with auto-fluorescence of F_420_, unique to methanogens, aggregated in the corrosion products (Figure 3). If CH_4_ was produced by hydrogenotrophic methanogenesis (CO_2_+4H_2_→CH_4_+2H_2_O) from H_2_ generated by abiotic iron corrosion, the amount of methane produced was calculated to be 10,736 ppm. This amount was 10 times smaller than that of CH_4_ produced by the e-GW1 culture. Therefore, methanogens in the e-GW1 culture could uptake more electrons from Fe^0^ than from H_2_ generated by abiotic iron corrosion. In the e-GW2 culture, a decrease in SO_4_ was observed, while H_2_ and CH_4_ generation were not detected. The amount of H_2_ generated by abiotic corrosion (145.7 μmol-e^−^ equivalent) was insufficient to support the amount of SO_4_ reduction (632.3 μmol-e^−^−equivalent) by SRB in e-GW2. The results demonstrate that methanogens and SRB take up electrons from iron granules as electron donors induced corrosion in the e-GW1 and e-GW2 cultures, respectively.

#### 3.2.2. Isolation of Microorganisms with EMIC Activity

**Figure 4:**
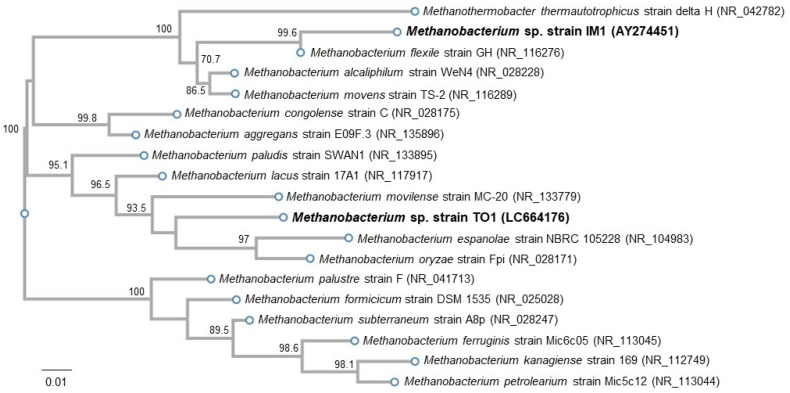
Phylogenetic tree showing the position of strain TO1 amongst other species of the *Methanobacterium* genus based on 16 S rRNA gene sequences. The TO1 *Methanobacterium* strain in this study, and the IM1 strain previously reported as EMIC methanogen, are shown in bold style. Numbers at branch points are percentages supported by bootstrap evaluation. Numbers in parentheses are GenBank accession number. Bars, 0.01 substitutions per position.

We isolated methanogens with strong EMIC activity from the e-GW1 sample, which was serially diluted and used for enrichment on freshwater basal medium agar plates under H_2_/CO_2_. Colonies formed on the agarose plates were picked and transferred to a freshly prepared medium containing iron granules to confirm the corrosion activity. The culture showed corrosion activity was cultured repeatedly for the isolation of a single colony. After repeated sub-culturing, methanogen from the e-GW1 sample was isolated. The isolated methanogen was 97.1 and 96.37 % identical to the *Methanobacterium movilense* strain MC-20 and *Methanobacterium oryzae* strain Fpi, respectively, based on its 16 S rRNA gene sequences (1348 bp). Corrosion activity of the *Methanobacterium* strain MC-20 and strain Fpi has not been reported; therefore, this isolate was named the *Methanobacterium* sp strain TO1. The phylogenetic tree strain TO1 among the other *Methanobacterium* species showed the position of strain TO1 was different from strain IM1, previously reported as EMIC methanogen [4], and the non-corrosive methanogen strain Mic6c05 isolated from iron tubercle on interior surface of pipeline transporting natural gas [21] (Figure 4).

**Figure 5:**
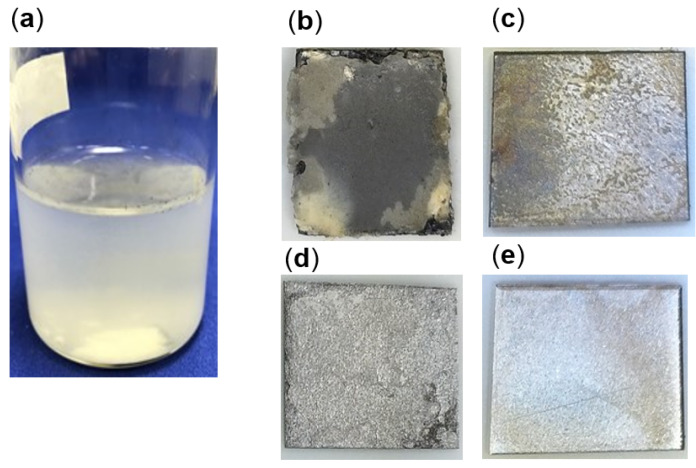
After cultivation of strain TO1 with carbon steel (**a**); carbon steel after cultivation of strain TO1 (**b**) and the abiotic control (**c**); carbon steel surface after the growth of strain TO1 and (**d**) the abiotic control (**e**) after washing with the acid solution.

**Figure 6:**
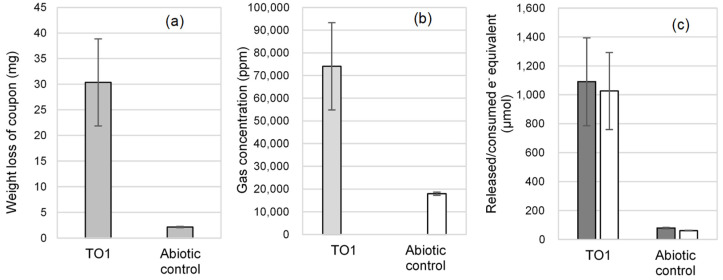
Corrosion activity of strain TO1 after 2 months of cultivation: (**a**) weight loss of carbon steel coupon, and (**b**) gas content in the headspace (light gray bar (CH_4_), white bar (H_2_)); (**c**) electron balance between electrons released from iron oxidation (dark gray bar) and electrons consumed by gas (CH_4_ and H_2_) generation (white bar).

To quantify the corrosion activity, strain TO1 was cultivated in freshwater basal medium with a carbon steel coupon instead of iron granules. After 2 months of cultivation, white corrosion products were observed in the TO1 culture (Figure 5a). White corrosion product was loosely deposited on a carbon steel coupon and dispersed in the culture medium when the coupon was picked. Under white corrosion, a grayish product was firmly attached to the coupon surface. Many troughs were observed on the metal surface under a gray crust in the TO1 culture (Figure 5b,d) but not in the abiotic control (Figure 5c,e). The weight loss of the coupons, H_2_ and CH_4_ in the headspace, and sulfate ion concentrations, were measured (Figure 6a,b). Based on the coupon weight loss, strain TO1 showed corrosion of 14 times higher at 0.36 mm/yr (30.4 mg ± 8.5 mg) than the abiotic control (0.025 mm/yr, 2.2 mg ± 0.13 mg), thus, indicating that *Methanobacterium* sp. TO1 could induce corrosion. CH_4_ production was observed at 74 061.2 ppm in the 38.8 mL headspace of TO1 culture after 2 months. No sulfate loss was detected. In contrast, CH_4_ was not detected and H_2_ accumulated at 17,911.7 ppm in the headspace of the abiotic control. Electron balances between electrons released from iron oxidation and that consumed by gas generation (CH_4_, H_2_) were calculated using Equations (1), (2) and (4) for the culture of strain TO1 and abiotic control (Figure 6c). In the culture of strain TO1, the electron amount released from iron was 1090 µmol e^−^ equivalents, which was comparable to that consumed for methane production by strain TO1 (1026.3 µmol e^–^ equivalents). This indicated that almost all electrons were used for methanogenesis of strain TO1. Meanwhile, abiotically generated H_2_ was equivalent to 62.1 µmol e^−^, which was not sufficient for methanogen observed in culture of strain TO1. More electrons (about 17 times) were needed for CH_4_ production observed in the TO1 culture than those for abiotic corrosion. Therefore, additional electrons were estimated to be extracted by strain TO1 from Fe^0^.

### 3.3. Electrochemical Incubation and CV Measurement of Methanobacteirum sp. TO1

**Figure 7:**
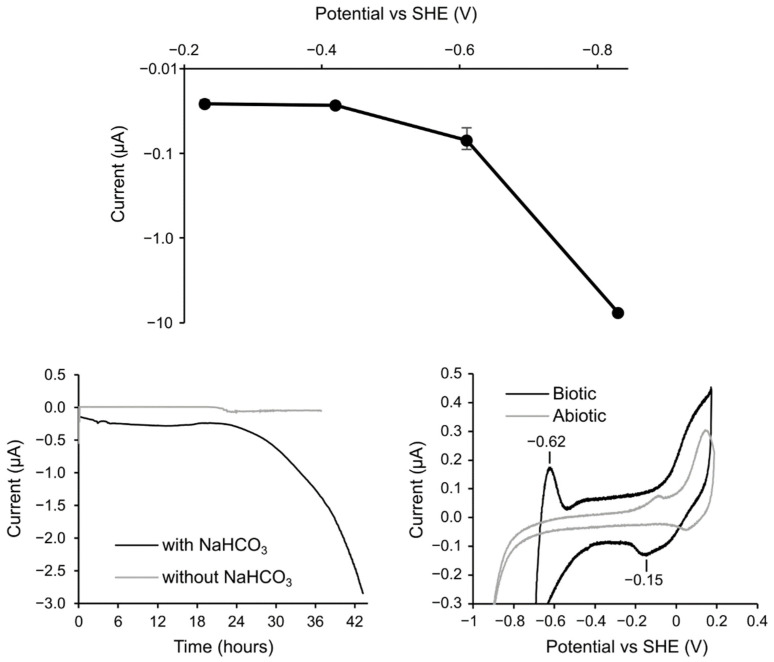
(**a**) Cathodic current production after electrical incubation for 110 h poised at different potentials with −0.23V, −0.42V, −0.61V, and −0.83V vs SHE in the presence of NaHCO_3_. An error bar represents standard deviation from two individual experiments. (**b**) Amperogram of cathodic current generation in the presence and absence of NaHCO_3_ at −0.85V vs SHE. (**c**) Cyclic voltammograms after electrical incubation at −0.83V vs SHE in the presence and absence of cells.

Electrochemical techniques were used to investigate the EMIC mechanism of the isolated *Methanobacteirum* sp. TO1. We first measured the cathodic current production at different electrode potentials to identify the electrode potential required to couple extracellular electron uptake with methanogenesis (Figure 7a). While the current was negligibly low at −0.23 V and −0.42 V vs. SHE, the current increased 2.6 and 280 times at −0.61 V and −0.83 V, respectively, after 110 h incubation. Therefore, we fixed the electrode potential at −0.83 V for electrochemical incubation. The time profile of cathodic current production at −0.83 V is shown in Figure 7b. The cathodic current began to increase from approximately 24 h and increased gradually until it reached a maximum of −2.85 μA after 43.3 h. In contrast, the cathodic current did not increase in the absence of NaHCO_3_, suggesting that the microbially captured electrons were used for CO_2_ reduction to methane (Figure 7b). To examine whether the electron uptake was associated with electron or hydrogen uptake, CV measurements were conducted after electrical incubation at −0.83 V in the presence of NaHCO_3_ (Figure 7c). The oxidative peak at −0.62 V and large catalytic cathodic current with onset around −0.5 V was observed mainly in the presence of *Methanobacterium* sp. TO1 cells. Given that a sharp increase in cathodic current assignable to H_2_ generation evolution was observed in nearly −0.9 V of cells, the cathodic current increase in the presence of cells is not assignable to hydrogen evolution but to microbial electron uptake. While a reductive peak was not detectable due to the large catalytic current, redox species with the oxidative peak at −0.62 V is attributable to the microbial electron uptake pathway. Its redox potential would be more negative than −0.62 V, which is 200 mV more negative than the potential of outer-membrane cytochrome identified in SRB *Desulfovibrio ferrophilus* IS5 enriched on iron as a sole electron donor [4,9]. The positive potential region showed that a redox peak pair with a middle potential of around −0.2 V is too positive to couple with the catalytic reaction. These data suggest that the Methanobacterium sp. TO1 has an electron uptake pathway with a negative redox potential that couples with methanogenesis.

## 4. Discussion

To enrich microorganisms capable of utilizing Fe^0^ as the sole electron donor from freshwater samples, we used iron granules as the sole electron donor and NaHCO_3_/CO_2_ as the sole carbon source. Using iron granules with a large surface area, SRB and methanogens were markedly enriched in groundwater samples. Because the e-GW1 sample showed a higher corrosion activity by methanogens than the e-GW2 sample, further enrichment and isolation were performed using the e-GW1 sample. The corrosive isolate from the e-GW1 sample was closely related (97.01% identity) to the hydrogenotrophic methanogen *M. movilense* strain MC-20 [18,22], isolated from the anoxic sediment of subsurface lake, based on its 16 S rRNA gene sequence. No corrosion activity was observed in related methanogens with strain TO1. In contrast, *Methanobacterium* sp. TO1 showed only 94.62% identity with *Methanobacterium* sp. IM1 reported as an EMIC methanogen [4]. The phylogenetic tree among the genus *Methanobacteirum* also showed the TO1 strain to be phylogenetically different from *Methanobacterium* sp. IM1. Cultivation experiments using carbon steel have demonstrated that *Methanobacterium* sp. TO1 can grow and produce methane by using iron as a sole electron donor. The amount of methane produced by strain TO1 cannot be sufficiently supported by the amount of abiotically produced H_2_ during cultivation. Therefore, these suggested that strain TO1 can uptake electrons from Fe^0^ and induce corrosion in a manner consistent with EMIC.

In general, methanogens induce mild corrosion at a low corrosion rate between 0.02 and 0.065 mm/yr [21] and produce non-conductive carbonate-based products, such as siderite, forming a protective layer against corrosion [23,24]. However, *Methanobacterium* sp. TO1 induced carbon steel corrosion with a high average corrosion rate (0.36 mm/yr) under the static condition in the serum bottle; the corrosion rate was higher than that of *Methanobacterium* sp. IM1 under static conditions (0.15 mm/yr) and was as high as the average corrosion rate of *Methanobacterium* sp. IM1, which was obtained in the flow system of a versatile multiport column filled with carbon steel beads [25]. The accumulation of non-conductive corrosion products, such as siderite formed by methanogenic corrosion, restricts the access of cells or enzymes to the iron surface and results in a lower corrosion rate. Because the accumulation of corrosion products on the iron surface is less under flowing conditions than under static conditions, strain TO1 may show a higher corrosion rate under flowing systems, such as natural environments, than that observed in this study. 

To validate the ability of strain TO1 to take up electrons from a solid surface, electrochemical incubation and CV measurements were performed. Electrochemical analysis revealed that strain TO1 could obtain electrons from carbon electrodes at lower than −0.61 V and utilize redox-active components with oxidation peak at −0.62 V for extracellular electron transfer. For only a few methanogens, the redox-active component that takes up extracellular electrons was electrochemically analyzed. For example, the EMIC methanogen, *Methanobacterium* IM1 can tightly attach to the electrode and utilize electrons from cathodes at -0.4 V through direct electron transfer. In contrast, the redox-active component for electron transfer was not identified [26]. Recently, *Methanosarcina barkeri* used two pathways to gain electrons from the electrode. The higher potential pathway was hydrogen production for methanogenesis by extracellular enzymes, such as hydrogenase (mid-potential: −279 mV) attached to the electrode, consistent with *M. maripaludis* [27,28]. Another lower-potential pathway is supported by an unidentified hydrogenase-independent mode with a potential −484 mV [29]. The electrochemical properties analyzed in this study showed that strain TO1 retrieved electrons from the electrode using a redox-active component over 200 mV, a lower potential than those of other previously reported methanogens. Thus, indicating that strain TO1 has a different mechanism for the uptake of electrons from other methanogens.

The ecological implication of the function of taking up electrons from Fe^0^ of methanogen underground is unclear. Fe^0^ is not a natural substrate for strain TO1 because almost all Fe^0^ is produced by human activity and is introduced into the environment. Recently, it has been reported that (semi) conductive iron-oxide minerals, such as magnetite, create an electrical connection between electrogens (e.g., iron-reducing bacteria) and methanogens [30]. Interspecies electron transfer through semi-conductive minerals has been shown to facilitate methanogenesis in natural soils and sediments [31,32]. The ability to utilize electrons from the solid-phase by strain TO1 makes it survive in environments with low available organic components and electron donors/acceptors, such as underground. Even if strain TO1 is a minor microorganism in natural groundwater, the TO1 strain would be enriched and increase the risk of EMIC when GW1 contacts the iron structure. The introduction of carbon steel increases microbial abundance and diversity in groundwater communities [33]. The discovery of TO1 provides a unique opportunity to assess the relative importance of the EMIC in freshwater environments. Further investigation of the EMIC mechanisms of methanogens is essential to develop diagnostic and control methods for EMIC caused by methanogens, particularly in freshwater environments without sulfate.

## Figures and Tables

**Table 1:** Chemical analysis of water samples collected in this study.

**Sample Name**	**pH**	**ORP** **(mv)**	**TOC** **(mg/L)**	**COD** **(mg/L)**	**Cl** **(mg/L)**	**NO_2_** **(mg/L)**	**Br** **(mg/L)**	**NO_3_** **(mg/L)**	**SO_4_** **(mg/L)**
GW1	8.4	71.8	2.9	4.4	10.1	ND	0.15	ND	10.5
GW2	7.1	−18.9	4.4	15.2	7.5	0.022	ND	0.28	14.2
RW	8.4	14.7	2.3	2.5	5.7	0.012	0.002	1.89	18.4
SW	7.9	−107.2	3.8	9.8	19,198.2	ND	69.4	ND	2628.6
**Sample Name**	**Fe** **(mM)**	**Li** **(mg/L)**	**Na** **(mg/L)**	**NH_4_** **(mg/L)**	**K** **(mg/L)**	**Mg** **(mg/L)**		**Ca** **(mg/L)**	
GW1	41	ND	23.5	1.2	7.6	4.5		14.3	
GW2	32	ND	9.1	3.0	2.8	3.1		7.5	
RW	5	ND	9.2	0.54	1.7	4.1		15.3	
SW	23	1.78	10,293.4	ND	543.3	1303.5		395.5	

## Data Availability

Not applicable.
